# Rubiaceæ

**Published:** 1868-02

**Authors:** D. L. Phares

**Affiliations:** Newtonia, Miss.


Vol. VIII. FEBRUARY, 1868. No. 12.

### ORIGINAL COMMUNICATIONS.

### ARTICLE I.

*Rubiaceæ:* By D. L. Phares, M. D., Newtonia, Miss.

This order contains about 250 genera—some of them of much importance in commerce, medicine, or the arts. In the Southern States we have 22 indigenous, and several exotic genera; among the latter, I may be permitted to mention the beautifnl everygreen shrub, Gardenia florida, (Cape Jessamine) with its very fragrant flowers. In this order are comprised also the Cinchona, (the various species of Peruvian bark) the Cephaelis Ipecacuanha, the Coffea Arabica, &c. The farmers of Florida and Southern Texas should give the Coffee plant a very careful and faithful trial as a field crop; and they would no doubt find it very remunerative. In this paper, I propese to notice very briefly only the medicinal plants of the order found in the Southern States.

Suborder I—Coffeeæ.

I. Rubia—(*Ruber*, red.) Madder.

R. tinctorum, (R. tinctoria, L.) This plant has been introduced and cultivated to a limited extent in some of the Southern States, and its culture could be much extended with profit to the farmer. It is a very important commercial article, the root furnishing almost every shade of red, purple, orange, yellow and brown dye, to almost black, according to the different modes of treating it. Taken into the stomach, it reddens the milk, urine and bones; the bones nearest the heart, and of young animals most. Medicinally, it was much employed by the ancients, and is noticed by Hippocrates, Galen, Dioscorides, and others, as valuable in jaundice, visceral affections generally, and all conditions requiring a diuretic. Many moderns have tried it. Cullen and others deny that it has any medicinal properties whatever. Home, B. S. Barton, Dewees, and others, commend it highly as an emmenagogue. When given at the near approach of the menstrual period, Dr. Dewees assures us that it is safer and more useful than any other emmenagogue medicine. He directs a pint of boiling water poured on an ounce of the powered root and a scruple of bruised cloves, and gently simmered fifteen minutes, of which, when cool and strained, the dose is a wineglassful every three or four hours. The dose of the powdered root is ʒ ss–ij. every three or four hours. I have not tried it in my own practice, but I have no doubt of its value in a recent state and prepared at a moderate temperature under cover. The Hindu practitioners employ R. cordifolia as an aperient, emmenagogue, and in cases of scanty or suppressessed lochia.

II. Galium.

This genus was noticed in the January number of this Journal—7th volume.

III. Cephalanthus. (*Kephale*, head, and *anthos*, flower.)

C. occidentalis. Globe Flower, Button Bush, Elbow Bush, Pond Dogwood, Bois de Marais, Americanische Weissbau, &c. This shrub is common, and is found in low wet places shallow ponds, and on banks of streams. The whole plant is bitter, but the bark of the root is the most valuable part. It is diuretic, aperient, pectoral, tonic, febrifuge. By the aborigines and subsequently by the whites it was used with much success in intermittent and remittent fevers. But it seems to be valuable mainly in cases of obstinate chronic cough, with emaciation, in which other remedies exert little or no beneficial effect. Many remarkable cures of cases of this kind have occurred under its use in decoction, syrup, or tincture. My own experience with it is limited. For decoction, use two ounces to the pint of water; the dose being ℥ i–ij. three to six times a day for cough; every two hours during the intervals of intermittent. The syrup, or ticture may be made of any convenient strength, and the dose proportioned as above.

### IV. Mitchella.

M. repens. Partridge Berry, Goose Berry, Chicken Berry, Deer Berry, One Berry, Winter Clover, Squaw Vine, &c. This little plant is evergreen, the stems and older leaves lying flat on the ground, and in many places forming a complete carpet. The leaves are roundish, ovate, flat, coriaceous, dark green, often mottled, shining above, sometimes purple beneath; the corals usually within, two in each ovary; the double drupe crowned with the calyx teeth of the two flowers; fruit edible, pleasant, rather incipid, scarlet. The plant is abundant in the forests, and is found from 28° to 69° North latitude. The whole plant is medicinal, yielding its virtues to water and alcohol. It is astringent, diuretic, tonic, nervine. Its action is directed specially to the genito-urinary organs. It is used with good results as a diuretic in rheumatism, gout, dropsy and suppression of urine. It is very valuable as a tonic, astringent and sedative in Gonorrhæ, gleet and prostatorrhœa, curing speedily many of these cases without the assistance of any other remedy. It is useful in dysuria and diarrhœa. Its soothing astringent properties render the aqueous extract valuable in treating excoreations, chaps, sore nipples, &c. It is an excellent uterine tonic. frequently order the hot infusion, or decoction, as a vehicle for Dr. Falk's Tinctura Antacrida, so much launded some years ago by the late Dr. Fenner, of New Orleans, in the treatment of dysmenorrhœa and lues venerea. It is greatly esteemed among the Indians, who use it for some weeks be fore confinement as a partûs praparatio. I have used it much for this purpose. It acts as a soothing, uterine tonic, allays nervousness, cramps and other unpleasant symptoms, and seems to put the whole parturient apparatus in the best condition for labor, by using internally three or four times a lay for three or four weeks before confinement. I have many times prescribed it where ladies were delicate, nervous, excitable, and have previously had difficult, painful, tedious labors and slow recoverys—always with happy results. In some of these cases where there is very great nervousness and uneasiness about convulsions, I order with this medicine some preparation of cimicifuga. The infusion of Mitchella makes a pleasant tea of which I direct from two to six ounces thrice daily, using an ounce of the drug to a pint of boiling water.

I feel a peculiar interest in this modest little plant, as the first, which, in my boyhood, on my first solitary botanical excursion into the forests, with Eaton's "Manual" in hand, I analysed and determined. As I have by many trials and much experience become better acquainted with its properties, and especially with the benefits it has conferred on "God's best, best gift to man," my favorable regard for it has grown not a little.

V. Chiococca. Cahinca, Cainca, Snow Berry.

C. racemosa. This plant is found in South Florida; but the roots found in the shops are obtained from the West Indies and South America. The root is the part used, though the bark is the only valuable portion. It has a disagreeable odor and a coffee-like taste, degenerating into a nauseous pungency. It is tonic, diuretic, emetic, purgative. In large doses it is a violent emetic and drastic purge. In small doses it stimulates the circulation, relaxes the bowels, promotes diuresis and diaphoresis. In Brazil it is used as a remedy for bites of poisonous reptiles, though another species growing there is prepared for this purpose. It is reputed useful in osteocepus, syphilis, rheumatism, amenorrhœa and dropsy. In the last, several practitioners have used it successfully in many cases. Like other drastic purgatives, we might expect it to induce sometimes the catamenial discharge. In its effects it resembles Senega a little, and Apocynum much more. It is a powerful agent and requires further careful trials; which it is hoped it will have in the hands of the physicians of Florida. The dose in powder is gr. xx to lx., but it may be given in tincture, decoction, or extract gr. x–xxx. It requires more than simple infusion to extract its properties.

### Suborder II—Cinchoneæ.

VI. Pinckneya. Georgia Bark, Bitter Bark, Fever Tree.

P. pubens is found in South Carolina, Georgia and Florida, along marshy banks of streams. It is closely allied to Cinchona, and is a bitter tonic. The bark has been long used in domestic practice with reported success in intermitted fever. Dr. Law, many years ago, reported seven cases in which he used it, and that in six it was perfectly successful. It did not distress the stomach even in doses of one ounce. It may be used in all respects as Cinchona, especially the pale, which it closely resembles in properties and action, and for which it is regarded by some as an excellent substitute. A full report on its properties and value as a remedial agent from personal observation by some of our Georgia friends would be very acceptable, or from any other source.

### VII. Exostemma.

E. Caribæum is found in South Florida. Several foreign species are known in commerce and have bitter febrifuge bark. The capsules of this species before quite ripe, are very bitter, and the juice causes a burning, itching of the lips. The bark is febrifuge, and when fresh emetic. The taste at first mucilaginous, sweetish, becomes afterwards bitter and disagreeable. This plant is worthy of further trial·

*Psychotria* lanceolata and P. undata, found in South Florida, should be examined, as the tropical species, P. emetica, has the same properties, and in about the same proportion as Ipecacuanha.

Some species of *Randia* also grow in South Florida, and may be found, on trial, as valuable as the R. dumetorum, which the Hindoo physicians consider their best emitic. They use the bark and pounded meat. The bruised fruit is employed also for intoxicating fish.

Several species of *Oldenlandia* are found from North Carolina southward to the Gulf of Mexico, and west to the Mississippi river. In India, the O. umbellata is used in pectoral affections; and some of our indigenous species might prove even more valuable.

### Suborder III. Loganieæ.

### VIII. Spigelia. Pink Root, Carolina Pink, Indian Pink, Worm Grass.

S. Marilandica is found in all the Southern States, and is probably more extensively used than any other anthelmintic. The whites learned its properties from the aborigines. When fresh it is very certain and efficient anthelmintic; but it deteriorates rapidly. As a vermifuge and narcotic its action is very similar to that of Melia. As a sedative febrifuge it, in some degree, resembles the Gelseminum. It is, however, so generally known and treated of in the books as not to require further notice here.

### IX. Gelsemium. Yellow Jessamine.

G. Sempervirens. In No. 534, Med. and Surg. Reporter, May 25, 1867, I have given a pretty full account of this very valuable medicine. By an oversight I did not mention its value in Gonorrhœa. As a vermifuge it acts much like Spigelia.

In fevers it seems to me not merely to counteract the effects of the "miasm," or fever poison by reducing the heat, force of the circulation, &c.; but, by acting directly on the same nerve centres attacked by the poison, to protect them; or rather, meeting the enemy at these points, to neutralize antidote or kill the poison itself. We know that sulphite of soda prevents fermentation, and when already in vigorous progress, instantly arrest it at any stage; as I, in common with others, have often proved both within and without the living body, seeming to kill the fermenting principle. Hence, its value fn dyspepsia, intermittents, etc. So Gelsemium, for besides its apparent antidotal properties in fevers, its action in the stomach is, in some respects, similar to that of sulphite of soda. Let me illustrate with a single case.

A gentleman having spent some months in the low lands of Louisiana, passing through a neighborhood where Cholera was prevailing, returned to this vicinity sallow and in bad condition generally. After a few days he was violently seized; vomiting and purging "rice water" by gallons, literally gallons, the discharges occurring every few minutes; legs severely cramped; extremities and surface icy cold; skin shrivelled, blueish; pulseless; eyes rolled back and glossy; speedy dissolution impending; opiates and other approved remedies had been used without effect, Such was the state of the case when Dr. J. H. Phares arrived. Having made the necessary examinations, he informed the medical friend in attendance that he could guarantee an arrest of the discharges with two doses of medicine; possibly, but not probably with one. Half a teaspoonful of tinct. Gelsemium and half a grain of morphine were given, and soon rejected, though some effect was noticed. As soon as the stomach was emptied, the second dose, f ʒ ; tinct. Gelsem. and gr. i. morphine, was given and completery arrested vomiting and alvine discharges. All unfavorable symptoms rapidly subsided and the case under control. But for the Gelsemium, it is almost certain that this case must have had a fatal termination.

In this instance was the action of the medicine on the nervous centres? It is hardly probable. The fluids, in these cases, all seem to rush into the alimentary canal to find an exit; and hence absorption from the stomach is difficult and impracticable. The cholera poison is said to act locally, directly on the alimentary tube. Did the Gelsemium, meeting the cholera sporules, animalcules, zymotic cells, or whatever else they may be called, there, at the very seat and centre of their attack, kill and anihilate them? Such at least seems to have been the process in this and many other cases which we have seen.

I would like to say much more, but this paper is already too long. Let it suffice for the present that having used this medicine some thousands of times, I ought to know something of its properties and effects; and I am fully convinced that it is one of the most valuable agents of the Materia Medica. For the benefit of those who have not seen the article above cited, I will add in a summary manner that Gelsemium is valuable in *fevers*, *rheumatism*, *neuralgias*, pulmonitis, pleuritis, hysteria, *dysmenorrhœa*, amenorrhœa, *gonorrhœa*, prostatorrhœa, chronic epilepsy, convulsions, paralysis, &c. Of course it is not equally valuable in all these affections, nor always the best remedy for some of them. A recent writer in a Western Journal undertakes to prove that it is worthless in all, by citing among other authors, Dodonæus! Gaspard Bauhein!! and Dioscorides!!! an anachronism of only 9600 years! An error in diagnosis, and hence the error in treating the subject—a mistake of Jasminum officinale which these writers notice, for Gelsemium, which they do not, and for obvious reasons could not mention.

The fresh roots should be used as it deteriorates in drying. Cut into small pieces and add ℥ iv. to spirit frument—or dilute alcohol oi; mascerate till the tincture when held up in a vial to the light, shows a violet tint; the deeper violet the better. Of this the dose is gtt x to f ʒ i, or more according to the special indications of the case.

